# Pair-bond strength is consistent and related to partner responsiveness in a wild corvid

**DOI:** 10.1098/rspb.2024.2729

**Published:** 2025-02-05

**Authors:** Luca G. Hahn, Rebecca Hooper, Guillam E. McIvor, Alex Thornton

**Affiliations:** ^1^ Centre for Ecology and Conservation, University of Exeter, Penryn TR10 9FE, UK; ^2^ Department of Biosciences, Swansea University, Swansea SA2 8PP, UK; ^3^ Centre for Research in Animal Behaviour, University of Exeter, Exeter EX4 4PY, UK; ^4^ Department of Behavioral and Cognitive Biology, University of Vienna, Vienna 1030, Austria

**Keywords:** corvids, pair bonds, responsiveness, social cognition, social intelligence hypothesis, social relationships

## Abstract

The need to maintain strong social bonds is widely thought to be a key driver of cognitive evolution. Cognitive abilities to track and respond to information about social partners may be favoured by selection if they vary within populations and confer fitness benefits. Here we evaluate four key assumptions of this argument in wild jackdaws (*Corvus monedula*), corvids whose long-term pair bonds exemplify one of the putative social drivers of cognitive evolution in birds. Combining observational and experimental behavioural data with long-term breeding records, we found support for three assumptions: (i) pair-bond strength varies across the population, (ii) is consistent within pairs over time and (iii) is positively associated with partner responsiveness, a measure of socio-cognitive performance. However, (iv) we did not find clear evidence that stronger pair bonds lead to better fitness outcomes. Strongly bonded pairs were better able to adjust hatching synchrony to environmental conditions but they did not fledge more or higher quality offspring. Together, these findings suggest that maintaining strong pair bonds is linked to socio-cognitive performance and may facilitate effective coordination between partners. However, they also imply that these benefits are insufficient to explain how selection acts on social cognition. We argue that evaluating how animals navigate trade-offs between investing in long-term relationships versus optimizing interactions in their wider social networks will be a crucial avenue for future research.

## 1. Introduction

Many social animals form differentiated social relationships or bonds, which can be defined operationally as repeated affiliative interactions between individuals in close proximity [1]. These bonds can entail the exchange of commodities such as social support [2], food [3], information [4] and grooming/preening [5] and may generate important fitness benefits [6,7]. For instance, numerous studies across taxonomic groups indicate that individuals that form strong social bonds and are well integrated within social networks show elevated survival or reproductive success [6–8]. The formation and maintenance of these strong bonds may be facilitated by abilities to track and respond to partners’ behaviour and make strategic social decisions [9–11]. Thus, the advantages of being able to establish strong social bonds could, in principle, generate selection on information processing, or cognitive abilities [10,12].

These ideas are encapsulated by the highly influential but controversial Social Intelligence (or Social Brain) Hypothesi*s* (SIH or SBH; [13–17]); for critiques, see [18–27]. While variants of the hypothesis differ in their emphasis (e.g. on ‘Machiavellian’ manipulations [15], social learning [28] or cooperation [29]), they share a common focus on the importance of navigating social relationships as a central driver of cognitive and brain evolution. For example, many primates form multiple stable relationships [30]. Accordingly, there is evidence that primate species that live in bigger groups (with a greater number of potential social partners) tend to have bigger brains ([16,18,31]; but see [27]), which is assumed to reflect greater cognitive abilities [16]. The fundamental logic of the SIH may be extended to other taxa. For instance, like primates, some birds—most notably corvids and parrots—are highly social and renowned for their large brains and sophisticated cognitive abilities [32,33]. However, avian brain size appears to be associated not with the quantity of social connections [22,26,34,35] but with long-term pair bonding ([26,36]; although again this finding is controversial [22]). Some authors therefore argue that pair bonding imposes selective pressures on cognition [26]. This bird-focused version of the SIH (sometimes known as the Relationship Intelligence Hypothesis [26]) posits that in long-term pair bonds with interdependent fitness outcomes, partners must track information about one another to minimize conflict and enable effective cooperation. This is assumed to generate substantial cognitive demands but lead to increased reproductive success. The benefits of such ‘relationship intelligence’ are therefore suggested to be a key driver of cognitive evolution in birds [26,36].

To date, investigations of the SIH have relied largely on comparative analyses of neuroanatomy and behaviour, often with contentious and contradictory results [20,24,27]. Moreover, the assumption that neuroanatomical measures such as brain size are suitable proxies for cognitive abilities is questionable [37,38]. Thus, there is growing recognition that if we are to understand the potential for selection to act on cognitive traits, approaches focusing on the causes and consequences of within-species variation are also necessary [19,39,40]. If bond strength varies within populations but is consistent within individuals and dyads and leads to increased fitness, then cognitive abilities underlying the formation and maintenance of strong social bonds could come under positive selection. Bond strength can be broadly conceptualized as the extent to which social partners engage in affiliative behaviours, which may reflect the value, compatibility and security of the relationship [41]. To quantify bond strength, different species-specific measures have been used, including time spent in proximity, affiliation (e.g. grooming, preening) and food sharing (e.g. [42,43]). In birds, there is evidence that pair-bond strength varies between pairs in captive [44–48] and wild [49] populations. Evidence for consistency of pair-bond strength is limited to between-year repeatability of spatial proximity in wild greylag geese (*Anser anser*; [49]) and within-year stability of affiliative ‘clumping’ in captive zebra finches (*Taeniopygia guttata*; [48]). Studies also suggest that the duration of pair bonds is positively linked to reproductive success (e.g. [50,51]). For instance, captive cockatiels (*Nymphicus hollandicus*) with stronger bonds have increased reproductive output compared with those with weaker bonds [52], and captive zebra finches with more stable bonds start their breeding attempts before those with less stable bonds [48]. Pair-bond strength could lead to fitness benefits by allowing pairs to better coordinate their behaviour in response to fluctuating environmental conditions. To understand whether and how selection acts on relationship strength in natural contexts, fitness outcomes must be investigated in wild populations. Moreover, despite being a foundational assumption of the SIH [10], the link between social bonding and cognition remains unclear. Indeed, in principle, interacting repeatedly with the same partner(s) could reduce uncertainty and allow partners to pool their skills, thus reducing cognitive demands [53–55]. Conversely, information processing abilities that enable individuals to detect and respond to a partner’s state could facilitate the maintenance of successful cooperative relationships [26,56]. To evaluate these possibilities, an important step is to examine whether individual socio-cognitive performance is positively associated with the maintenance of strong social bonds. Quantifying cognitive performance in this context is challenging, particularly in the wild, but one promising approach is to focus on variation in responsiveness to partners. Given that cognition, by definition, involves information processing [12], the ability to track and respond to information from social partners (or ‘social competence’ [57]) can provide a tractable proxy for socio-cognitive performance.

Here, we tested four key assumptions of the SIH within one study system, wild jackdaws (*Corvus monedula*), a highly social corvid species. Like other corvids, jackdaws have large brains and exhibit social behaviours including social information use [9,58], social support [26] and cooperation [9,59]. Crucially, jackdaw societies are centred around long-term, genetically monogamous pair bonds [42,60–63]—the key putative social drivers of cognitive evolution in birds [26,36]. As cavity nesters, they also take readily to nest boxes, facilitating monitoring of social behaviour between partners and reproductive success. By monitoring social behaviour within nestboxes, we quantified pair-bond strength during the nest-building and incubation stages of the breeding season (April to June). We selected these stages because partners showed greater affiliative behaviour than in other phases (e.g. when adults are focused on provisioning chicks), allowing us to quantify pair-bond strength and its variation among pairs. For natural selection to act, traits must be variable across individuals and stable over time. We made the underlying assumption that an individual’s ability to form and maintain social relationships is reflected by the observable strength of the pair bond [26]. We therefore predicted (1) that pair-bond strength should vary between pairs and (2) be consistent within pairs, that is, repeatable between observations both within and across years. Furthermore, if bond strength and cognition are linked, we predicted (3) that individuals that maintain stronger pair bonds should be more responsive to information about their partner’s affective state (i.e. show greater ‘social competence’ [57]). To test this, we used measures of responsiveness from an experiment where females were exposed to a stressor in the absence of their male partner. We treat males’ responses as proxies for their cognitive ability to detect and respond to information about their partner’s distress [64]. Finally, we examined the adaptive value of maintaining strong pair bonds using records of breeding attempts across years where conditions varied substantially. Specifically, more asynchronous hatching is thought to be advantageous in resource-poor years because the brood size is quickly reduced, thus increasing the probability that a small number of chicks survive rather than entire brood failure ([65,66], but see [67]). Conversely, more synchronous hatching is thought to be advantageous in resource-rich years [65]. As the control of hatching asynchrony depends on coordination between the female, who incubates, and the male, who provisions his incubating partner [61], pairs with stronger bonds may be better able to optimize hatching asynchrony to match current environmental conditions. We predicted (4) that variation in pair-bond strength should be linked to the pair’s ability to respond to changing environmental conditions and, ultimately, to their reproductive success. Specifically, we expected that pairs with stronger bonds would (i) be better able to adjust breeding attempts to current conditions through changes in hatching asynchrony and (ii) successfully rear more fledglings, with greater overall fledgling mass.

## 2. Methods

### (a) Subjects and study sites

Behavioural, morphometric and breeding data were collected from colour-ringed jackdaws during the breeding seasons in 2014, 2015, 2018 and 2019 at three nest box colonies in Cornwall, UK. The study sites were the University of Exeter’s Penryn campus (Site X: 50°10’23’ N, 5°7’6’ W), a churchyard and adjacent fields in Stithians (Site Y: 50°11’26’ N, 5°10’51’ W) and a private farm near Stithians (Site Z: 50°11’56’ N, 5°10’9’ W). Data from 125 individuals (63 females, 62 males; imbalance owing to repairing) were included in this study. Further details of the study population, including breeding data and capture and ringing methods, are in the electronic supplementary material (§1.1.).

### (b) Video data

During the breeding season, from March to June, the pair cooperate to build the nest [59,61] and the female incubates the eggs while her partner brings her food [61]. We captured video footage of jackdaws inside their nest box during the nest-building and incubation stages of the breeding seasons. We recorded footage in the early morning, starting shortly after sunrise, using hidden CCTV cameras (electronic supplementary material, §1.2).

We transcribed footage using a detailed behavioural ethogram for fine-scale quantification and analysis of pair-bond strength (electronic supplementary material, table S1). We focused on the nest-building and incubation stage because (variation in) affiliative behaviour between partners is particularly prevalent in those phases (L.G.H., R.H. 2020, personal observation). It is worth noting, however, that social behaviour and bond strength may differ outside of these contexts. During the nest-building stage in 2018 and 2019, we recorded 142.18 h of footage across 54 videos (mean length = 2.63 ± 1.13 h per video) for 39 pairs. Of those 39 pairs, 11 pairs were recorded during the nest-building stage in both 2018 and 2019 (in five instances a pair was recorded twice within one nest-building stage). During the egg laying/incubation stage in 2014, 2015, 2018 and 2019, we recorded 362.24 h of footage across 132 videos (mean length = 2.74 ± 1.07 h per video) for 65 pairs. Of those 65 pairs, 34 pairs were recorded during the incubation periods of at least 2 different years (in 21 instances a pair was recorded twice within one incubation stage). All 39 pairs that were recorded in the nest-building stage were also recorded in the incubation stage, meaning that the remaining 26 pairs were only recorded during the incubation stage. The video footage that we analysed varied in length owing to occasional technical issues and changes in light conditions impacting the visibility of colour rings to identify birds.

### (c) Data processing and statistical analysis

We conducted all data processing and statistical analysis in R v.4.0.2 ([68]; see electronic supplementary material,§1.3., for details).

### (d) Quantifying pair-bond strength

Behaviours recorded from nest box videos were standardized by (i.e. calculated as a proportion of) the lengths of the videos. To quantify pair-bond strength and identify interrelationships between potential affiliative behaviours ([43,47,69]; electronic supplementary material, figure S1), we used principal components analysis (PCA; package: *psych* [70]; see electronic supplementary material, §§1.3 and 2.2). For the PCA, we considered the following behaviours previously hypothesized to be important affiliative behaviours between bird partners: ‘food-sharing’ (specifically the male providing food to his female partner; [3]); ‘contact’ [42], ‘allopreening’ [5], ‘time together’ [45] and ‘copulation’ [47]; (see electronic supplementary material, table S1). We also included ‘male visit rate’ in the incubation stage because females must remain in the nest box to incubate, but the rate at which the males visit their partner may vary and may be correlated with other affiliative behaviours. Finally, we included ‘chatter’, a distinctive call that partners often make when together at the nest box. Allopreening was split into ‘male-initiated’ and ‘female-initiated’ for the nest-build stage but not at the incubation stage because almost all allopreening (94.55%) was male-initiated. The first principal component, PC1, explained a substantial proportion of variation (electronic supplementary material, figure S3) in the data for both the nest-building (43.3% of variation explained by PC1; dominated by allopreening, contact and time together; electronic supplementary material, figure S2a, table S2) and incubation stage (59.5% of variation explained by PC1; dominated by male chatter, female chatter, allopreening, contact and time together; electronic supplementary material, figure S2b, table S2). We therefore used each pair’s PC1 value as the measure of ‘pair-bond strength’ in both instances when constructing statistical models.

### (e) Statistical modelling

We checked model residual plots (using the package *DHARMa* [71]) to ensure model assumptions were met [71]. For cases where we built competing models, we compared models using Akaike’s Information Criterion (AIC; [72]). If models differed in AIC by two or more, we selected the model with the lowest AIC. We identified influential points as any datapoints more than four times the mean Cook’s distance. We ran each final model both with and without influential points. If results from full models and models without influential points differed, we report the results of both models. No final models were over-dispersed or zero-inflated and all showed acceptable model fit.

#### (i) Does pair-bond strength (1) vary between pairs, and is it (2) consistent within pairs?

We tested whether pair-bond strength within pairs remained consistent over time, using repeatability analysis in the package *rptR* [73]. Specifically, we tested whether pair-bond strength was repeatable within years and between years for both the nest-building and incubation stages. We first ran repeatability models with no covariates to obtain an unadjusted estimate of repeatability of pair-bond strength. Following this, we controlled for covariates and obtained an adjusted repeatability estimate. Covariates for the within-year models (for both stages) were days since the female’s fertile period and the time of day that the video started. Where pair-bond strength was found to be repeatable within years, we then calculated a mean value of pair-bond strength per year and used this value to estimate repeatability between years. For between-year models, we included the age of the male and year as covariates. We used ‘male age’ as a proxy for the time the pair had been together because this might impact bond strength as well as its repeatability and consequences for reproductive success. The age of partners is typically strongly correlated because jackdaws tend to form their pair bond early in life (during their first winter) with other individuals within their cohort ([61]; G.E.M unpublished data). We did not include days since the female’s fertile window and video start time as covariates owing to the use of the mean value of within-year pair-bond strength as the response variable. In all models, we log-transformed pair-bond strength (Gaussian distribution). For each model, parametric bootstrapping (*N*_boot_ = 1000) quantified uncertainty, while significance testing was implemented using likelihood ratio tests (LRT) and through the permutation of residuals (*N*_perm_ = 1000).

#### (ii) Is pair-bond strength related to partner responsiveness?

In 2019, we ran a field experiment to test whether males responded to their partner’s distress [64]. To do this, we used playbacks to expose incubating females (*n* = 27) to the sound of a foreign male at the nest box—an important stressor as foreign males subject nesting females to violent attacks [60,64]. Focal females’ male pair-bonded partners were absent from the area during playbacks and therefore blind to the stressor. We supplemented experimental data with data from natural forced extra-pair copulation events (*n* = 6) where we had a measure of male behaviour towards his female partner both pre- and post-stressor, and the male was absent for the stressor (experimental and natural data produced qualitatively the same results). In this original study we found that, overall, males altered their behaviour after their partner had experienced the stressor, indicating that they responded to their partner’s state [64]. However, the nature and magnitude of changes in male behaviour were highly variable [64]. While the overall effect was that males reduced visit rates and affiliation (suggesting that males use indicators of female state to minimize their own exposure to risk), some males showed the opposite effect and a few did not change their behaviour at all. Here, we therefore measured ‘partner responsiveness’ as the absolute change in male-initiated direct affiliative behaviour (contact and allopreening) towards his partner for 1.5 h pre-stressor and 1.5 h post-stressor (the time period over which the strongest effects were found in the original study). We focused on the absolute difference, rather than the direction of behavioural change, because we were interested in the degree to which males responded to their partner’s state, rather than whether the response was to increase or reduce affiliation (either of which could provide benefits, depending on the context [64,74]). In our model, partner responsiveness—the dependent variable—was log-transformed for improved model fit (Gaussian distribution). We included pair-bond strength (during the incubation stage), data type (experimental or natural), whether the male returned before the female, and minimum age of the male (which resulted in a better model fit than minimum number of years together) as covariates for the full model. We followed this analysis with two further models to test: first, we examined whether female behaviour changed as a function of pair-bond strength, and thus whether males could simply have been responding to the differential magnitude of female behavioural change. Second, we also tested whether males were responding to overt changes in female behaviour, in other words, whether males’ change in affiliative behaviour was more pronounced when females themselves showed a greater magnitude of behavioural change in response to the stressor.

#### (iii) Do pairs with stronger bonds have higher reproductive success?

We constructed generalized linear mixed models (GLMMs) to test whether pair-bond strength (during the incubation stage) was linked to fitness outcomes (*n* = 93–95 data points from *n* = 47–48 pairs). Because pair-bond strength was repeatable during the incubation stage of the breeding season (see §3), we tested whether pair-bond strength measured in this stage was associated with reproductive outcomes. For each response variable reflecting one aspect of reproductive success (see *Success in fledging offspring*), we tested competing models that included either ‘minimum number of years together’ or ‘minimum male age’. We always included ‘pair-bond strength’ (per video), ‘male tarsus’, ‘female tarsus’, ‘lay date’, ‘year’ and the ‘rate of male provisioning during female incubation’ (i.e. ‘food-sharing rate’; uncorrelated with affiliative behaviours in our PCA yet potentially important for reproductive success) as fixed effects. Tarsus length, used as a proxy for skeletal body size, was previously found to be associated with reproductive success [75]. Furthermore, we included ‘pair ID’ and ‘site’ as random effects. For all fitness outcomes, we compared models with linear and quadratic pair-bond strength to test for directional and stabilizing selection on pair-bond strength [76]. We also compared models with and without an interaction term between pair-bond strength and year. We tested this interaction because how selection acts on behaviour can vary according to environmental conditions (e.g. [77]). All GLMMs were fitted with a gamma distribution except for one model, which was fitted with a binomial distribution (proportion of hatchlings that fledged as a response variable).

#### Coordination of hatching synchrony

Daily observation allowed us to monitor the date of egg hatching in order to calculate hatching synchrony. Hatching synchrony was calculated as the date of the last hatch minus the date of the first hatch, divided by the total number of eggs that hatched. We examined whether a strong pair bond may facilitate fitness-enhancing responsiveness to changing environmental conditions across breeding seasons by testing the relationship between hatching synchrony as a response variable and an interaction between year and pair-bond strength. In jackdaws, females incubate their eggs while being provisioned by their partner [78]. The synchronicity of hatching depends on the female’s incubation behaviour [79], which in turn is influenced by the male’s provisioning behaviour [78]. Hatching synchrony may therefore be related to how well a pair is able to coordinate their behaviour in the face of environmental variability. To explore this further, we also constructed a model examining the relationship between hatching synchrony as a response variable and partner responsiveness (males’ responsiveness to distress in female partners, as above) as a predictor variable (site and pair ID were included as random effects).

#### Success in fledging offspring

The measures for fledging success that we tested for each pair (per year) were (i) number of fledglings, (ii) total mass of fledglings and (iii) proportion of hatched chicks that fledged. Because of low levels of variance in the number of fledglings per year (72.87% of *n* = 47 pairs fledged two or three offspring), we also tested the cumulative number of fledglings per pair over 5 years. This required sub-setting the data to pairs for whom we had 5 years of reproductive success data (*n* = 12). For this model, pair-bond strength was calculated as the mean value of pair-bond strength per pair across all available data points in the incubation stage. To test how reproductive success varied across years for the whole population, we also examined the relationship between year and population-wide number and mass of fledglings. For these models, we included ‘year’, ‘male and female tarsus length’, ‘minimum male age’ or ‘years together’ and ‘lay date’ as predictor variables. We included ‘pair ID’ as a random effect, but site was not included owing to convergence issues. These models allowed us to gain insight into whether some years appeared particularly difficult, most likely owing to limited resource availability. To test whether hatching synchrony interacted with year to influence reproductive success, we ran GLMMs with (i) number of fledglings, (ii) total mass of fledglings and (iii) proportion of hatched chicks that fledged across the entire population as response variables and hatching synchrony as a predictor. However, models (1) and (2) would not converge with the inclusion of site, so it was removed. For each response variable, we compared model performance with and without an interaction term between year and hatching synchrony. Comparing population-level reproductive success and hatching synchrony across years based on potential environmental changes allowed us to evaluate whether variation in these outcomes was indirectly linked to variation in pair-bond strength via adjustments in hatching asynchrony across resource-poor and resource-rich years. Again, we also constructed models examining the relationship between measures of reproductive success (same as above) as a response variable and partner responsiveness (male responsiveness to distress in female partners as a predictor variable; site and pair ID were included as random effects).

## 3. Results

### (a) Does pair-bond strength (1) vary between pairs, and is it (2) consistent within pairs?

**Figure 1.**
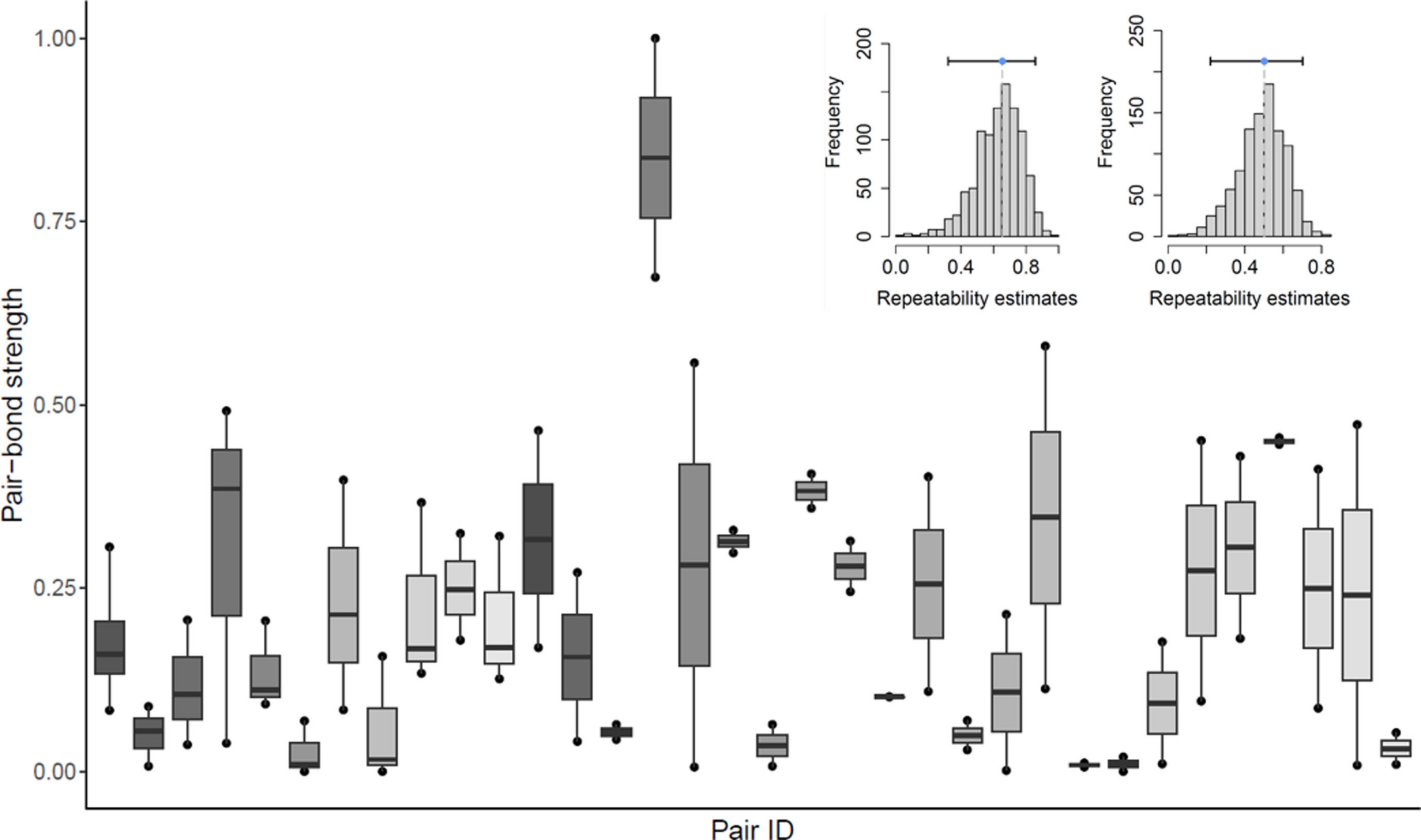
Pair-bond strength per pair, quantified during the incubation stage using PCA (including the following behaviours: time spent together in the nest box, male chatter, female chatter, allopreening and time spent in contact). Each datapoint represents the strength of a pair’s bond in a single year; thus, boxplots show the range of bond strength per pair across multiple years. Repeatability plots are depicted in the top right corner, displaying estimates (including confidence intervals shown as horizontal bars) of within-year bootstrap repeatability (left) and between-year bootstrap repeatability (right). Jackdaw pair-bond strength was significantly repeatable both within and between years, with confidence intervals not overlapping with zero, and the results were robust to the removal of a pair with extraordinarily high pair-bond strength. Note that for visualization purposes, pair-bond strength (log-transformed PCA values) is standardized between 0 and 1. PCA, prinicipal component analysis.

Pair-bond strength varied considerably between pairs both during the nest-building and the incubation stages (figure 1). Pair-bond strength was not repeatable in the nest-building stage (see electronic supplementary material, §2.4). But during the incubation stage (for which sample sizes were higher), pair-bond strength was highly repeatable (adjusted repeatability including covariates) both within-year (*n* = 21 pairs that were recorded twice, adjusted *R* = 0.65, 95% CI [0.34, 0.87], *P*_perm_ < 0.01, *P*_LRT_ < 0.01) and between-years (*n* = 34 pairs for which measures were available from 2.35 ± 0.54 different years (mean ± s.d.), adjusted *R* = 0.50 , 95% CI [0.26, 0.71], *P*_perm_ < 0.01, *P*_LRT_ < 0.01; figure 1). There was one highly affiliative pair (evident in figure 1) but results were robust to their removal (within-year: adjusted *R* = 0.49, 95% CI [0.08, 0.80], *P*_perm_ = 0.03, *P*_LRT_ = 0.02; between-year: adjusted *R* = 0.29, 95% CI [0.03, 0.57], *P*_perm_ = 0.02, *P*_LRT_ = 0.05).

### (b) Is pair-bond strength related to partner responsiveness?

**Figure 2.**
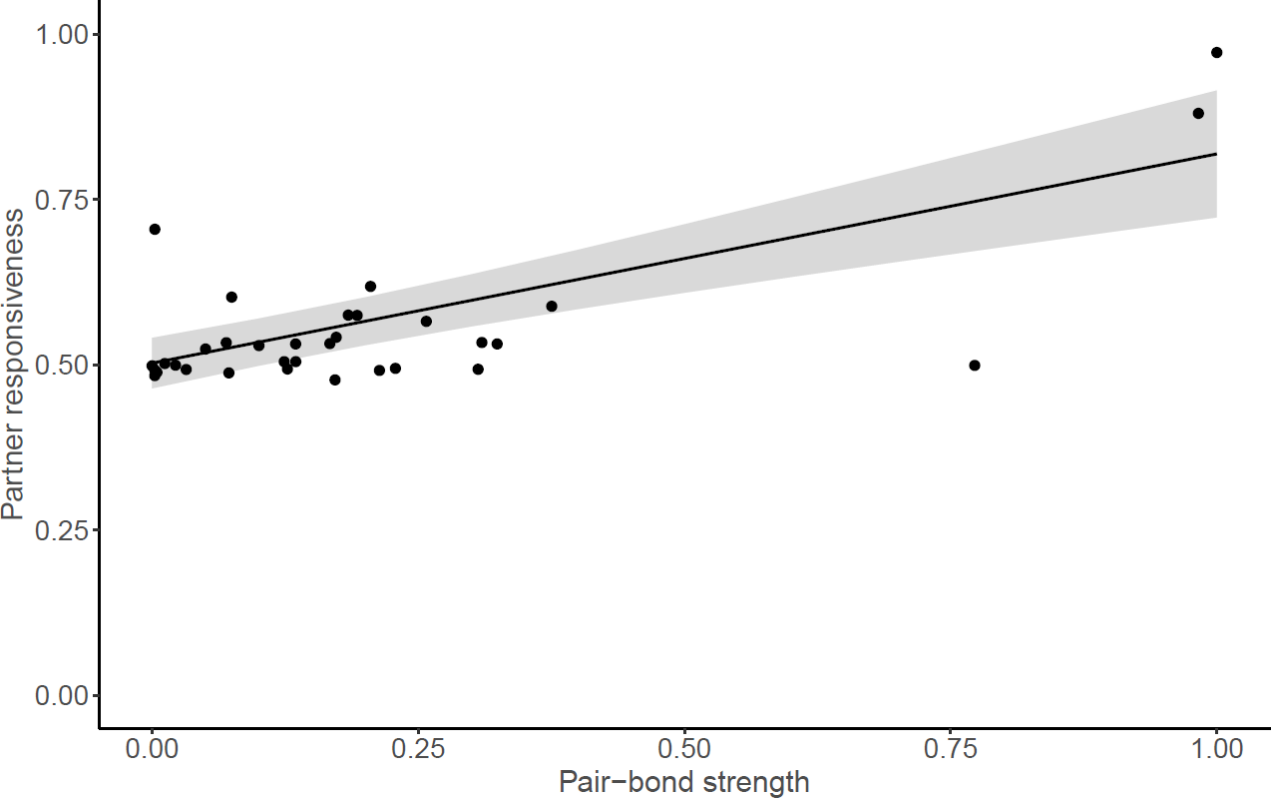
Pair-bond strength was significantly positively correlated with partner responsiveness (a measure of socio-cognitive performance in experimental tests), both in the full model and with influential points excluded. Note that for visualization purposes, pair-bond strength and partner responsiveness are standardized between 0 and 1 (influential datapoints with value above 0.75).

There was a significant relationship between pair-bond strength and the responsiveness of the male to his partner’s distress (figure 2), where males in stronger pair bonds showed a larger absolute positive change in behaviour following their partner experiencing a stressor (*N*_pairs_ = 33; *β* = 0.71, s.e. = 0.12, 95% CI [0.49, 0.94], *p* < 0.001). This result was robust to the removal of influential points (*N*_pairs_ = 30; *β* = 0.12, s.e. = 0.01, *χ*^2^ = 137.97, 95% CI [0.10, 0.14], *p* < 0.01). Partner responsiveness was not significantly associated with the other covariates included in the model (electronic supplementary material, §2.5). Previous analyses have reported weak evidence for slight declines in female chatter calls and begging rates post-stressor [64]. However, male responses were not linked to any detectable change in female behaviour: there was no significant relationship between the absolute change in female begging or chatter and pair-bond strength (begging rate: *N*_pairs_ = 24, *β* = −0.14, s.e. = 0.09, *χ*^2^ = 2.65, 95% CI [−0.31, 0.03], *p* = 0.10; chatter duration: *N*_pairs_ = 23, *β* = −0.15, s.e. = 0.15, *χ*^2^ = 0.95, 95% CI [−0.45, 0.15], *p* = 0.33) or the magnitude of the female’s change in vocalizations and the magnitude of the male’s behavioural change (begging rate: *N*_pairs_ = 23, *β* = 0.19, s.e. = 1.88, *χ*^2^ = 0.01, 95% CI [−3.50, 3.88], *p* = 0.92; chatter: *N*_pairs_ = 24, *β* = −0.56, s.e. = 0.86, *χ*^2^ = 0.42, 95% CI [−2.25, 1.13], *p* = 0.52).

### (c) Do pairs with stronger bonds have higher reproductive success?

#### (i) Coordination of hatching synchrony

**Figure 3.**
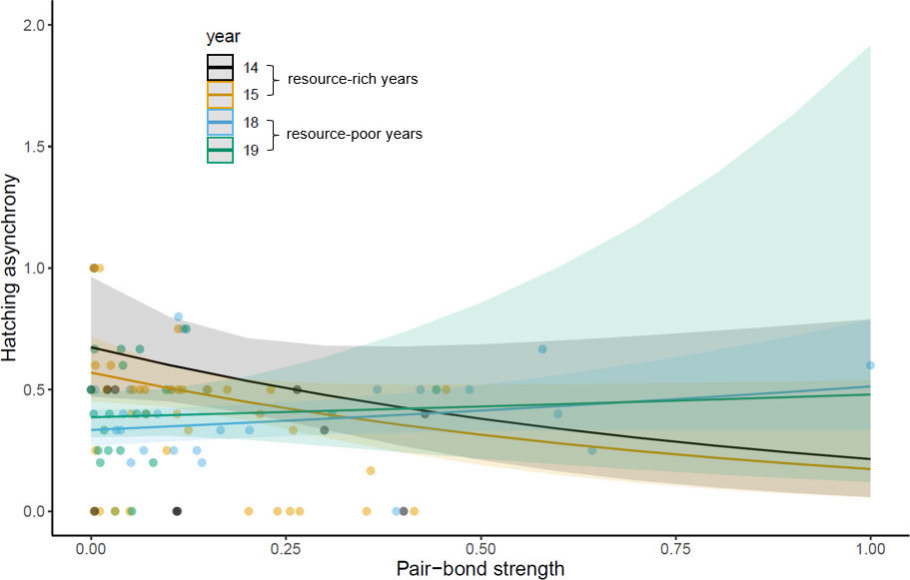
We found a significant interaction between pair-bond strength and year (a proxy of environmental conditions) on hatching synchrony. Here, a higher value on the *y*-axis represents more asynchronous hatching. Note that for visualization purposes, pair-bond strength (log-transformed PCA values) is standardized between 0 and 1.

The interaction between pair-bond strength and year was significantly associated with hatching synchrony (full model: *N* datapoints = 102, *N*_pairs_ = 50, *χ*^2^ = 8.00, *p* = 0.046; figure 3), although the model term only bordered significance with influential points removed (*N* datapoints = 92, *N*_pairs_ = 47, *χ*^2^ = 7.06, *p* = 0.07). Pairwise comparisons between years were consistent for the full model (2018 relative to 2015: *β* = 0.11, s.e. = 0.05, 95% CI [0.01, 0.21], *p* = 0.02) and the model with influential points removed (*β* = 0.22, s.e. = 0.10, 95% CI [0.03, 0.42], *p* = 0.03). The full model also revealed a significant difference in hatching synchrony between 2018 and 2014 (*β* = 0.22, s.e. = 0.08, 95% CI [0.06, 0.38], *p* < 0.01). There was no detectable relationship between hatching synchrony and males’ responsiveness to their partner’s distress (*β* = 0.04, s.e. = 0.03, 95% CI [−0.02, 0.09], *p* = 0.16).

#### (ii) Success in fledging offspring

**Figure 4.**
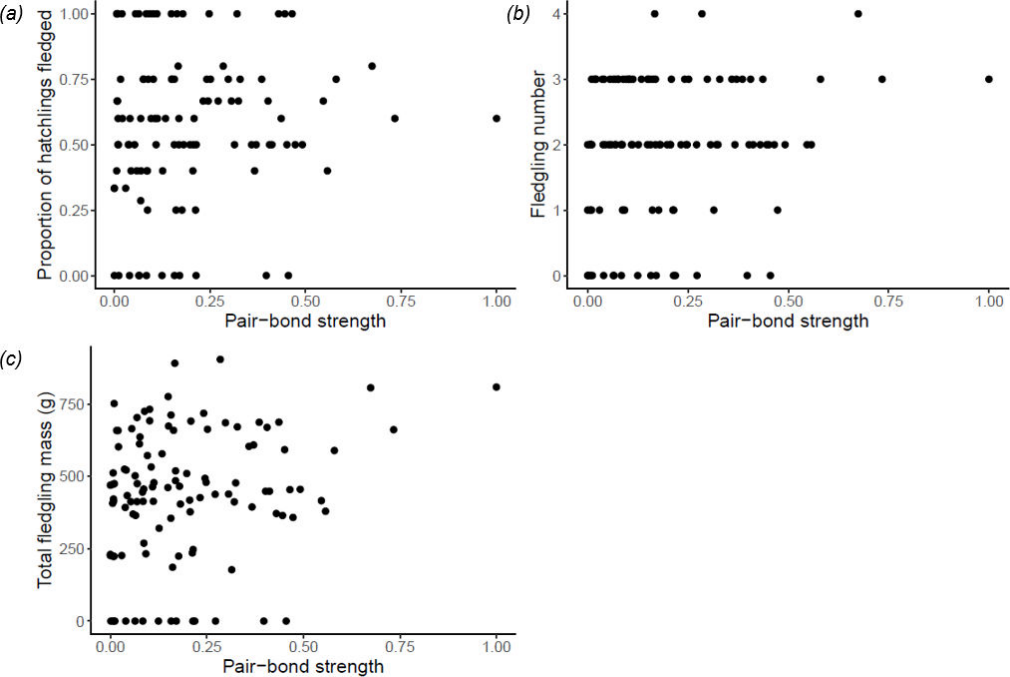
There was no significant relationship between pair-bond strength (mean pair-bond strength per pair per year) and different proxies of reproductive success, such as (*a*) the proportion of hatchlings that fledged, (*b*) the number of fledglings and (*c*) the total fledgling mass. Note that for visualization purposes, pair-bond strength (log-transformed PCA values) is standardized between 0 and 1.

Pair-bond strength was not associated with the number of fledglings (*N*_pairs_ = 47, *β* = 0.003, s.e. = 0.02, *χ*^2^ = 0.03, 95% CI [−0.04, 0.03], *p* = 0.86), mass of fledglings (*N*_pairs_ = 48, *β* = 0.02, s.e. = 0.02, *χ*^2^ = 0.73, 95% CI [−0.02, 0.05], *p* = 0.39), proportion of hatched chicks that fledged (*N*_pairs_ = 47, *β* = −0.01, s.e. = 0.04, *χ*^2^ = 0.04, 95% CI [−0.08, 0.06], *p* = 0.85) or cumulative fledging (*N*_pairs_ = 12, *β* = 0.11, s.e. = 0.14, *χ*^2^ = 0.65, 95% CI [−0.16, 0.39], *p* = 0.42; see electronic supplementary material, table S3 for details; figure 4). We find qualitatively the same results if responsiveness to partner distress, rather than pair-bond strength, is included as a predictor (see electronic supplementary material, §2.6). According to population-wide models of number and mass of fledglings per year, 2018 and 2019 were poor years for jackdaw reproductive success relative to 2014 and 2015 (figure 3; see electronic supplementary material, §2.6 for further details). All models without the interaction term between year and hatching synchrony were better than models with its inclusion (where a ‘better’ model has an AIC more than or equal to two less than the competing model; electronic supplementary material, table S4). This suggests that hatching synchrony did not interact with year to influence reproductive outcome, at least across the 4 years of our study.

## 4. Discussion

We examined four key assumptions of the SIH in pair-bonding birds. We found evidence that pair-bond strength (i) varied between pairs, (ii) was consistent within pairs and (iii) was positively associated with a measure of partner responsiveness. However (iv) although pairs with stronger bonds were better able to adjust hatching synchrony to current environmental circumstances, we found no evidence that pair-bond strength was associated with reproductive success.

### (a) Does pair-bond strength (1) vary between pairs, and is it (2) consistent within pairs?

In our jackdaw populations, pair-bond strength (1) varied between pairs and (2) was consistent within them during the incubation stage. Pair-bond strength was not repeatable during the nest-building phase, but this may reflect the more limited sample sizes. The repeatability estimates of pair-bond strength during the incubation stage in our study are higher than the average repeatability of behaviour in general (0.37) as reported in a meta-analysis of behavioural consistency [80] and similar to the repeatability of social network position (0.41–0.62) in wild great tits (*Parus major*; [81]). These results are consistent with the assumption that the ability to maintain strong bonds represents a stable trait that could come under selection [26]. While variation in social bonding could reflect a multiplicity of underlying factors, including endocrine profiles [82], individual personality [83] and familiarity [50], the SIH assumes a central role for information-processing abilities [26]. We turn our attention to this assumption in the next section.

### (b) Is pair-bond strength related to partner responsiveness?

Our results suggest that in more strongly bonded pairs, the male was more responsive to his partner’s distress. While males responded in variable ways to their partner following the partner’s exposure to a stressor (in both experimental and natural contexts; [64]), males in stronger pair bonds showed a larger absolute change in partner-directed behaviour (contact and allopreening behaviour; irrespective of the direction of behavioural change). Given that male responses were not linked to any overt changes in female behaviour, this indicates that males in stronger bonds are more responsive to very subtle cues of female state. This is reminiscent of findings in humans, where individuals who show better socio-cognitive performance (e.g. better recognition of and response to the emotional state of others) form stronger friendships [84]. However, although our findings imply an important role for cognition in the broad sense [12] because males must detect, process and act upon information about their partner’s current state (i.e. exhibit social competence; [57]), the specific cognitive mechanisms are unknown. One possibility is that responses are driven by emotional contagion, where information about a partner’s state leads to matching states between partners [56]. For example, there is evidence for emotional contagion from research on common ravens (*Corvus corax*) [85–87]. Elucidating the mechanisms underlying social bonding is complicated by the fact that bonds are a product of the behaviour of both partners. Evaluating reciprocal responsiveness by both partners is logistically challenging, especially in wild animals, but will be an important focus for future work. For instance, this could be achieved by conducting experiments that integrate behavioural and physiological methods (e.g. by using infrared thermography and playback experiments [88,89]). We also note that we cannot unequivocally rule out the possibility that weakly bonded males were just as capable as strongly bonded counterparts of detecting their partner’s state but were simply less motivated to respond. Nevertheless, we would argue that to understand the potential for selection to shape partner responsiveness (and the underlying proximate mechanisms), what matters is how information processing translates into action.

### (c) Do pairs with stronger bonds have higher fitness?

While our results provide no evidence linking pair-bond strength directly with proxies of reproductive success, they do suggest hat bond strength may be linked to variation in hatching asynchrony. Specifically, relative to weakly bonded pairs, more strongly bonded pairs hatched their clutches more synchronously in ‘good’ years and less synchronously in ‘poor’ years. Thus, the behaviour of strongly bonded pairs appears to match adaptive hypotheses on optimal asynchrony strategies, which suggest that more asynchronous broods are favoured in less productive years [65]. This indicates that pairs with stronger bonds may have been better able to adjust their hatching synchronicity to environmental conditions. The precise mechanisms through which more strongly bonded pairs may be better at adjusting hatching synchrony to environmental conditions are unclear. Hatching synchrony is, however, related to incubation initiation [79], which is under female control in jackdaws [63]. If males fail to respond to female incubation initiation cues and food-share with their partner, then incubation initiation will be disrupted because the female must leave the nest box in order to feed. Therefore, one explanation as to why more strongly bonded pairs are better able to adjust hatching synchrony to environmental conditions could be that males are more responsive to their partner’s behaviour and thus can better coordinate the initiation of incubation. In this study, our measure of partner responsiveness was not directly linked to hatching synchrony and measures of reproductive success. However, this measure (i) had a lower available sample size than pair-bond strength and (ii) was taken in response to the partner’s distress and not in response to a behaviour related to breeding, such as incubation. Further research will be needed to better characterize partner responsiveness across contexts and understand its adaptive significance. In theory, the adjustment of hatching synchrony to environmental conditions should result in fitness benefits [65,66], but we detected no signal that hatching synchrony interacted with year to influence reproductive success across the 4 years analysed. We also found no effect of pair-bond strength on the total number and mass of fledglings per pair per year or cumulative fledging success per pair over multiple years. The absence of a relationship between pair-bond strength and fitness is at odds with previous work showing that pair-bond strength correlates positively with fledging success in captive cockatiels [52] and that pair-bond duration, the stability of the bond and the familiarity of partners (likely to be facets of pair-bond strength) influence fitness outcomes [48,50,90–93]. Moreover, this result could be seen to undermine a central tenet of the SIH, suggesting that forming and maintaining strong relationships may not be a key driver of cognitive evolution in large-brained birds (cf. [26,36]).

Before drawing this conclusion, however, alternative hypotheses must be addressed. First, survival is a key component of fitness that we were not able to test in this study. In multiple species, social bonds have been linked to increased probability of survival for adults [7,94,95] and their offspring [6,96,97]. Investigating whether individuals in strong pair bonds have a higher probability of survival, or if their offspring have a higher probability of survival post-fledging, is a vital step forward in elucidating whether pair-bond strength influences fitness. Our finding that the rate of food sharing from the male to the female was negatively related to the number of fledglings produced raises the tentative possibility that food sharing within the pair during an earlier stage (which could enhance partner condition and survival) trades off against offspring provisioning at a later stage of the breeding attempt. Second, while the pair bond is the most valuable relationship in corvid society, pairs do not exist in a social vacuum but are embedded within wider social networks and navigate numerous other social bonds [26,98]. For instance, partners work together to interrupt relationship formation between potential competitors in ravens (*C. corax*; [99]), while in jackdaws and rooks (*Corvus frugilegus*) partners aid each other in fights against third parties [100] and learn from and associate with flock members independently of one another [42,101]. There is also some evidence that jackdaws with more central social network positions have better reproductive outcomes [75]. Given that time spent together is an important component of pair-bond strength, and that it takes time to monitor, form and maintain non-pair relationships, there is an implied trade-off between the management and maintenance of pair versus non-pair relationships. Indeed, in humans a trade-off can arise between the quality of a relationship with a romantic partner and the quantity of non-romantic social bonds [102,103], while in jackdaws we have found that investment in pair bonds trades off against the strategic adjustment of social associations in the wider social network [9]. Such trade-offs could limit strongly bonded partners’ access to valuable social and cultural information and so obscure the detection of a direct relationship between pair-bond strength and reproductive success. Studies examining how animals navigate the potentially conflicting demands of different types of relationships are now needed to characterize such trade-offs and deepen our understanding of the cognitive demands of sociality. Finally, while we have treated bond strength as a trait that may impact fitness, in reality the strength of social bonds is an emergent product of two (or more) interacting individuals. As recent advances in quantitative genetics highlight, the expression of social and cognitive traits of individuals can influence and be influenced by the phenotypes (and underlying genotypes) of other individuals [104]. Future research will therefore need to consider the impacts of interacting genotypes (indirect genetic effects; [104]) to better understand the strength and direction of selection shaping social bonding and the underlying cognitive processes.

## 5. Conclusion

The SIH (and its avian-specific formulation, the Relationship Intelligence Hypothesis) emphasize long-term, cooperative social bonds as central to understanding cognitive and brain evolution [26]. We found support for three key assumptions of the SIH in wild corvids, birds renowned for their sophisticated cognitive abilities: the strength of relationships varies between mated pairs of jackdaws, is consistent within pairs and is linked to a measure of responsiveness to partner state. However, while pairs with stronger bonds may be better able to coordinate reproductive behaviour in response to variable environmental conditions, we did not find any evidence that pair-bond strength influences reproductive success. In a field largely dominated by broad-scale comparative studies, further intraspecific testing of links between cognitive performance, social behaviour and fitness is vital to resolve debates as to whether and how social relationships may drive cognitive evolution. As a key part of these efforts, we also need experiments to determine the cognitive mechanisms underpinning responses to social partners. For instance, experimental manipulations under field conditions could allow us to test whether focal partners detect and respond to changes in the state of social partners (e.g. hunger, stress and vocal indicators of affect) across different ecologically relevant contexts. Finally, in this context we also propose that observational and experimental studies investigating how animals navigate trade-offs between long-term partnerships and wider social connections (e.g. by manipulating the outcomes of foraging associations [9]) are now vital to understand the cognitive demands of social life and their evolutionary consequences [55].

## Data Availability

Data and scripts are available at [105]. Supplementary material is available online [106].
